# Influence of Constipation in the Behavior of Circulating Alpha- and Beta-CGRP Levels in Chronic/High-Frequency Migraine Patients After CGRP Monoclonal Antibodies

**DOI:** 10.3390/biomedicines13051254

**Published:** 2025-05-21

**Authors:** Gabriel Gárate, Marcos Polanco, Jorge Madera, María Muñoz-San Martín, Marta Pascual-Mato, Vicente González-Quintanilla, Julio Pascual

**Affiliations:** 1 Service of Neurology, University Hospital Marqués de Valdecilla, Universidad de Cantabria and IDIVAL, 39008 Santander, Spain; gabriel.garate@idival.org (G.G.); marcos.polanco@scsalud.es (M.P.); jorge.madera@scsalud.es (J.M.); maria.munoz@idival.org (M.M.-S.M.); vicente.gonzalez@scsalud.es (V.G.-Q.); 2 Service of Gastroenterology and Hepathology, University Hospital Marqués de Valdecilla, Universidad de Cantabria and IDIVAL, 39008 Santander, Spain; marta.pascual@scsalud.es

**Keywords:** alpha-CGRP, beta-CGRP, chronic migraine, constipation, CGRP monoclonal antibodies, high-frequency episodic migraine, migraine

## Abstract

**Background/Objectives**: Migraines contain neurological and gastrointestinal manifestations. The first specific migraine preventive drugs, CGRP monoclonal antibodies (mAbs), though efficacious and very well-tolerated in general, induce constipation as their main adverse event. Our goal was to analyze the role of the two isoforms of CGRP in the development of constipation in patients treated with mABs. **Methods**: We prospectively measured by ELISA circulating alpha- and beta-CGRP levels in 133 high-frequency episodic/chronic migraine patients before and three months after mAbs treatment and correlated these levels with a number of clinical variables, including the development of constipation during this treatment. **Results**: Twelve patients (9.0%) noticed de novo constipation with mAbs. Demographics, efficacy end-points, profile of preventive treatment, and comorbidities, with the exception of anxiety/depression, were superimposable between patients with or without emergent constipation. Basal alpha-CGRP levels (49.5 [29.2–73.8] pg/mL) significantly decreased at month three of treatment (40.5 [20.4–61.0] pg/mL; *p* < 0.0001), both in patients with and without emergent constipation. Pre-treatment circulating beta-CGRP levels (4.0 [2.1–6.2] pg/mL) remained unchanged after three months of treatment (4.3 [2.5–6.0] pg/mL; *p* = 0.574) in the whole series but were selectively reduced in patients with emergent constipation (*p* = 0.034). **Conclusions**: This is the first work exploring the role of the two isoforms of CGRP in the pathophysiology of constipation with mAbs. Our results suggest that the antagonism on the alpha-CGRP isoform plays a relevant role in the antimigraine action of mABs but not in the development of constipation. By contrast, the specific reduction in beta-CGRP levels in patients with emergent constipation supports the role of beta-CGRP antagonism in the development of this adverse event.

## 1. Introduction

Migraines could be considered an example of a disorder involving the gut–brain axis. Gastrointestinal symptoms, such as nausea/vomiting, constipation, or diarrhea, are classical hallmarks of migraines and some gastrointestinal disorders, such as irritable bowel syndrome, celiac disease, or inflammatory bowel disease, which are more common in migraine subjects than in the general population. One further example of this gut–brain connection could be the COVID-19 disease, where migraine-like headaches and gastrointestinal symptoms are among the most frequent extra-respiratory symptoms [1,2,3].

**Figure 1:**
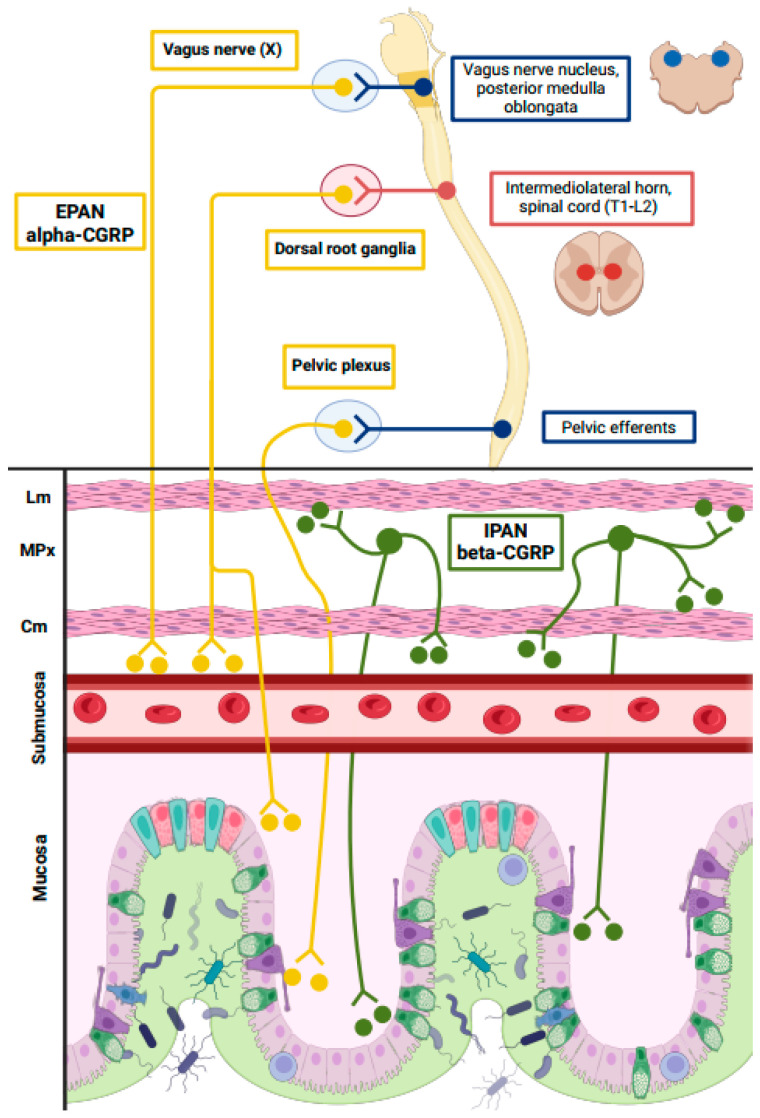
Schematic distribution of CGRP in the gastrointestinal tract. In yellow, extrinsic primary afferent neurons (EPANs) release alpha-CGRP. These neurons originate from the vagus nerve, the dorsal root ganglia, and the pelvic plexus, and their axons reach the intestinal mucosa and submucosa, as well as the submucosal blood vessels. In green, intrinsic primary afferent neurons (IPANs) release beta-CGRP. These neurons originate from the myenteric plexus (MPx) and reach the circular and longitudinal muscle layers (Cm and Lm, respectively), submucosal blood vessels, and intestinal mucosa.

This clinical interaction should be mediated by chemical transmitters able to exert actions in both organs, and CGRP has been one of the proposed involved molecules [4,5]. First, the two isoforms of CGRP are located both in the brain and the trigemino-vascular system as well as in the entire gut. In fact, CGRP-containing fibers originating in the pain and vegetative brain nuclei arrive at the myenteric plexus, where the extrinsic primary efferent neurons (EPAN) release CGRP, mainly in its alpha-CGRP isoform. The beta-CGRP isoform predominates in the gastrointestinal system, where it is released by the intrinsic primary afferent neurons (IPANs) (Figure 1) [6]. Second, CGRP manipulation in the human body has been shown to induce relevant gastrointestinal symptoms. Intravenous infusion of CGRP in human volunteers triggers migraine-like headaches together with diarrhea [7], and constipation is the most frequent adverse event of a CGRP block with CGRP antagonists [8]. While alpha-CGRP levels seem to play a key role in migraine pain pathophysiology, as they are increased in patients with active migraine and normalize when treated with CGRP monoclonal antibodies (mAbs), circulating beta-CGRP levels do not change in a series of patients with frequent migraine attacks versus healthy controls [9]. However, recent findings suggest that beta-CGRP could play an important role in the pathophysiology of gastrointestinal symptoms, as exemplified by their specific increase in COVID-19 patients with diarrhea [10], or by its reduction in early phases of inflammatory bowel disease (IBD) [11].

As stated above, constipation is the most frequent adverse event in patients treated with mAbs [8]. Our objective here has been to analyze the potential role of the two CGRP isoforms in the development of constipation in migraine patients treated with mAbs. With that aim, we prospectively determined circulating alpha- and beta-CGRP in a series of migraine patients before and after treatment with mAbs and correlated these levels with a number of clinical variables, including the development of constipation during this treatment.

## 2. Materials and Methods

### 2.1. Ethical Considerations

This work followed the ethical principles of the Declaration of Helsinki and was approved by the Ethics Committee of Investigations and Medications of Cantabria (Spain), and its approval was published in the record 28/2020 of 11 December 2020. All participants gave written informed consent for their inclusion in this study. Data collection and analysis were conducted using pseudonymized datasets to ensure confidentiality.

### 2.2. Study Design, Population, and Clinical Assessments

Patients who met high-frequency episodic or chronic migraine (HFEM/CM) criteria according to the International Classification of Headache Disorders (ICHD-3) [12], were older than 17 years, had no response/intolerance to at least 3 oral preventatives plus onabotulinumtoxinA in the case of CM, and began the use of mAbs were included in this study. Detailed clinical data of the participants, including date of migraine initiation and duration of an HFEM/CM situation, presence of aura, historical and current acute and preventive treatments, compliance with analgesic overuse (AO) criteria, and monthly headache and analgesic consumption days, were available at mAbs initiation. We also recorded comorbidities; the presence of constipation was self-recorded and diagnosed as less than 3 spontaneous bowel movements per week, straining, fecal blockage, or manual maneuvers for fecal extraction for at least 25% of defecations, majority of lumpy or hard stools, or use of laxatives to maintain stool frequency/consistency. Concomitant preventive drugs remained stable during the first three months of the study period and also for at least one month prior to mAb administration. We kept record of monthly headache days, monthly migraine days, analgesic consumption, adverse events, and Patient Global Impression of Change (PGIC) scale at the end of the third quarter. Patients were revised at 1 and 3 months after the first dose of mAb and fulfilled a headache calendar. At these two visits, patients were specifically asked about the presence of constipation. We considered constipation as an adverse event if it appeared de novo during mAb treatment or if baseline constipation had worsened. Constipation was arbitrarily graded as mild (no relevant influence on the quality of life of the patient and no or minor dietary changes required), moderate (required daily dietary changes and/or laxatives), and severe (mAb had to be stopped in spite of dietary changes and laxatives).

### 2.3. Blood Extraction and Laboratory Determinations

Patients with HFEM/CM had two blood extractions, one just before the initiation (basal) and three months after mAb treatment (M3). All samples were obtained on migraine-free days and having taken no symptomatic treatment in the previous 24 h. After a fasting period of at least 12 h, blood samples were extracted in the morning (9–12 a.m.), were left to clot for 10 min, then centrifuged at 3500 rpm and 4 °C for 10 min, immediately transferred into sterile tubes, and stored at −80 °C until assayed. Without exception, samples remained cryopreserved for less than 6 months before being analyzed.

Alpha- and beta-CGRP serum levels were determined using commercial enzyme-linked immunosorbent assays (ELISAs; Abbexa, Cambridge, UK, reference: 257902; CUSABIO, Wuhan, China, reference: CSB-E08210h, respectively) as reported by our group [13].

### 2.4. Statistical Analysis

Categorical variables are reported as percentages, whereas continuous variables are displayed as mean ± standard deviation (SD) for normally distributed data and as mean ± SD together with median with interquartile range (IQR) for non-normally distributed data. Normality assumption of quantitative continuous variables has been checked using the Shapiro–Wilk test. Evolution of continuous variables over the course of mAb treatment was assessed with Wilcoxon signed-rank test, as appropriate. Comparison of distribution of categorical variables was performed employing the chi-squared test.

All tests were two-tailed, and significance was set at *p*  <  0.05. GraphPad Prism version 9.4.1 (GraphPad Software, Boston, MA, USA) was the software utilized for all the conducted analyses.

## 3. Results

### 3.1. Patient Population, Response, and Constipation with mAbs

We included 133 migraine patients (mean age = 47.7 ± 10.7 years; range 19–69 years); 128 (96.2%) were women. One hundred and eleven (84.1%) met diagnostic criteria for CM and twenty-two (15.9%) for HFEM. Their main clinical characteristics are illustrated in Table 1. A total of 52 (39.1%) were treated with erenumab, 45 (33.8%) with galcanezumab and 36 (27.1%) received monthly fremanezumab. Eighty-one (60.9%) and eighty-seven (65.4%) showed at least 50% response in headache and migraine days, respectively. Basal monthly headache and migraine days (22.1 ± 7.1 and 18.5 ± 6.7, respectively) significantly (*p* < 0.0001) improved at month three (10.5 ± 8.4 and 8.1 ± 6.7, respectively). There was a significant reduction in the proportion of patients with analgesic overuse before (114; 83.5%) and three months after treatment with mABs (33; 24.8%; *p* < 0.0001).

A total of 24 migraine patients reported constipation during treatment with mABs; twelve had a previous history of constipation, which had not changed during this treatment. Therefore, 12 (9.0%) patients experienced de novo constipation with CGRP monoclonal antibodies: seven (13.5% of the patients on erenumab) with mAbs against receptor (erenumab) and five (6.2% of the patients on galcanezumab or fremanezumab) with mAbs against ligand (two with galcanezumab and three with fremanezumab).

**Figure 2:**
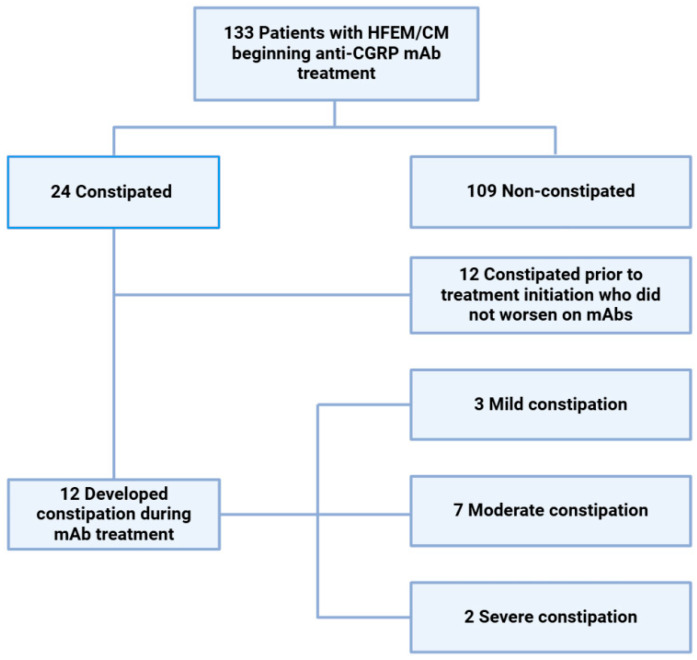
Flow chart of CM/HFEM migraine patients treated with mAbs participating in this study with/without constipation.

Constipation was evaluated as mild in three subjects, moderate in seven, and severe in two patients, who had to stop this treatment due to this adverse event after 3–6 months (Figure 2). Demographics, response rates, comorbidities, and profile of preventive treatment did not significantly differ between patients with or without emergent constipation, except for the presence of anxiety/depression, which had a higher prevalence in the constipation group and scoring in the PGIC scale, which was significantly worse in patients with de novo constipation (Table 2)

### 3.2. Alpha- and Beta-CGRP Levels

Basal alpha-CGRP levels (mean ± standard deviation: 57.4 ± 44.4 pg/mL; median [interquartile range]: 49.5 [29.2–73.8] pg/mL) significantly decreased at month three of treatment (47.8 ± 44.8 pg/mL; 40.5 [20.4–61.0] pg/mL; 16.7% mean decrease; *p* < 0.0001).

**Figure 3:**
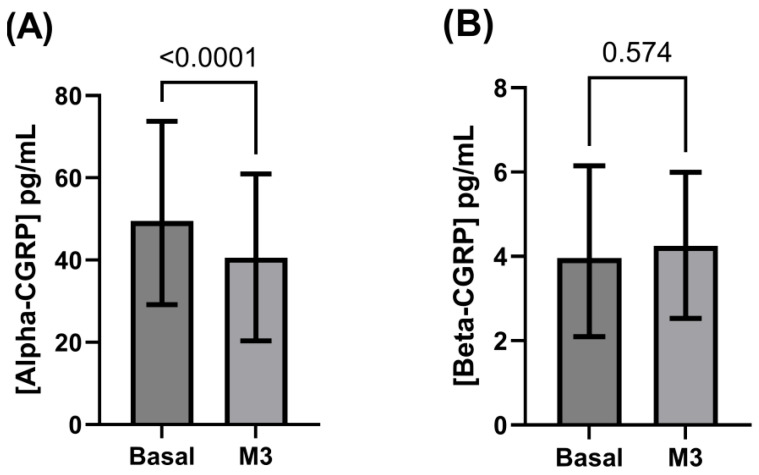
CGRP levels during mAb treatment in the whole series. (**A**) Significant decrease in alpha-CGRP levels after three months of treatment (M3) as compared to pre-treatment (basal) levels. (**B**) No significant change in beta-CGRP in M3.

**Figure 4:**
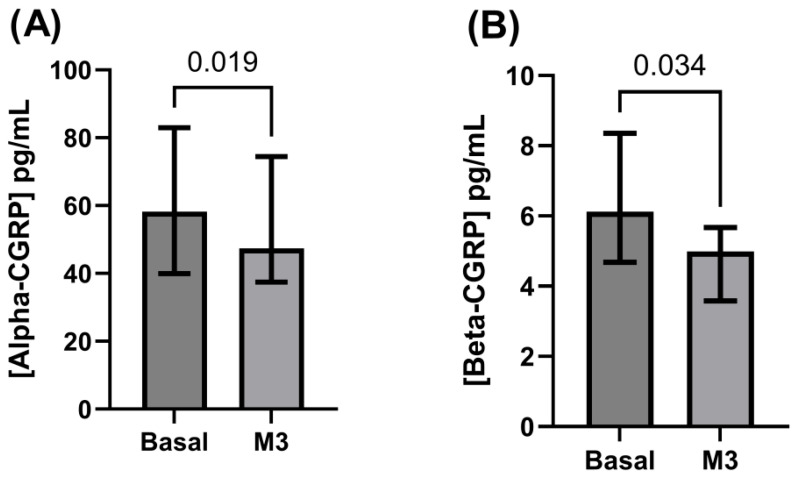
Behavior of CGRP levels in patients with emergent constipation during treatment with mAbs showing a significant decrease after 3 months of treatment (M3) both in alpha-CGRP (**A**) and beta-CGRP levels (**B**).

Pre-treatment circulating beta-CGRP levels (4.2 ± 2.7 pg/mL; 4.0 [2.1–6.2] pg/mL) remained unchanged after three months of treatment (4.4 ± 2.5 pg/mL; 4.3 [2.5–6.0] pg/mL; *p* = 0.574) (Figure 3). In those patients with no change in their intestinal rhythm after CGRP monoclonal antibodies (n = 121; 91.0%), alpha-CGRP levels were significantly decreased at three months (46.9 ± 45.3 pg/mL; 39.3 [19.6–60.7] pg/mL) versus baseline (56.2 ± 44.3 pg/mL; 48.0 [28.6–73.4] pg/mL; *p* < 0.0001), while beta-CGRP levels again did not change after 3 months of treatment (4.5 ± 2.6 pg/mL; 4.2 [2.5–6.0] pg/mL) compared to baseline (4.0 ± 2.6 pg/mL; 3.5 [2.0–6.1] pg/mL; *p* = 0.162). In the 12 patients complaining of constipation, basal alpha-CGRP levels (70.0 ± 43.4 pg/mL; 58.2 [40.0–83.0] pg/mL) were significantly higher when compared to those levels after 3 months (57.4 ± 35.6 pg/mL; 47.4 [37.5–74.5] pg/mL; *p* = 0.019). By contrast, circulating CGRP levels after three months of treatment with monoclonal antibodies (4.5 ± 2.3 pg/mL; 5.0 [3.6–5.7] pg/mL) were significantly reduced as compared to baseline concentrations (6.7 ± 3.1 pg/mL; 6.1 [4.7–8.4] pg/mL; *p* = 0.034) (Figure 4). In patients experiencing emergent constipation, basal alpha-CGRP levels were numerically but not significantly higher than those seen in the global series (*p* = 0.213) and in the subgroup of patients without constipation (*p* = 0.176), while beta-CGRP levels were numerically and significantly higher at baseline when compared to those found in the whole series (*p* = 0.004) and in patients without constipation (*p* = 0.002).

Although concomitant preventatives remained unchanged three months before treatment with mABs and during the first quarter of treatment with mABs, due to its capacity to induce constipation, we examined the potential influence of amitriptyline in beta-CGRP levels. There were 43 patients on amitriptyline, and 6 (14%) had constipation before treatment with mABs versus 6/90 (6.7%; *p* = 0.170) who were not taking amitriptyline. Beta-CGRP levels in patients were identical before treatment in the group on amitriptyline versus those who were not taking this drug (4.2 ± 2.5 pg/mL versus 4.3 ± 2.8 pg/mL; *p* = 0.857).

## 4. Discussion

To the best of our knowledge, this is the first work exploring the potential role of the two isoforms of CGRP in the pathophysiology of constipation as an adverse event of mABs in the preventive treatment of migraines. As expected [9], interictal serum levels of alpha-CGRP significantly decreased during mAb treatment. This decrease was parallel to migraine response end-points, reasonably explains the antimigraine action of mABs toning down a hyperactive trigemino-vascular system, and further supports an actual role of circulating alpha-CGRP as a biomarker of migraine activity. Contrary to the association between alpha-CGRP levels and headache activity, there was no correlation between alpha-CGRP levels and constipation. In fact, the decrease in circulating alpha-CGRP levels was equivalent in HEFM/CM patients versus those without emergent constipation, which suggests that the antagonism on alpha-CGRP isoform does not play a relevant role in the development of constipation.

Leaving alpha-CGRP aside, we had different results in circulating beta-CGRP levels. Concurring with our previous results [9], we could not measure a modulation of beta-CGRP levels after successful treatment with mAbs in our whole series, which indicates that, contrary to alpha-CGRP, the beta-CGRP isoform does not seem to play a crucial role in the pathophysiology of migraine pain. By contrast, circulating beta-CGRP levels significantly decreased in those patients with emergent constipation after mAb treatment. With the exception of a higher rate of anxiety/depression, we could not find a significant relationship between demographics, clinical characteristics, concomitant preventive treatments, or migraine standard efficacy measures and the development of constipation with mAbs, which concurs with the proposal of a mechanism independent of alpha-CGRP for this adverse event. Interestingly, PGIC was the only measure scoring significantly worse in patients who developed relevant constipation, suggesting a negative influence of this adverse event in the subjective, global response to these drugs. Our results match with the predominant location of each isoform, the trigemino-vascular system for alpha-CGRP and the gastrointestinal system for beta-CGRP [2,3], and support a specific role for beta-CGRP antagonism in the development of constipation. Having said that, one intriguing finding in this series was that baseline levels of beta-CGRP were higher in those patients who developed constipation during mAb treatment. We do not have a final explanation for these results, but one hypothesis could be that in this subset of patients, the maintenance of adequate gastrointestinal motility requires an extra beta-CGRP activity, thus they would be more sensitive to developing constipation when the signaling of the peptide is limited.

### 4.1. Pathophysiological and Clinical Implications

CGRP exerts multiple actions in the gastrointestinal tract, including motor, secretory, vascular, and immune functions [2,3]. Beta-CGRP has a predominant gut location; its concentration in the intestine is seven times higher than that of the alpha-CGRP isoform [14,15,16]; therefore, though most experimental studies do not differentiate between alpha- and beta-isoforms, it could be assumed that data about gut CGRP are most probably referring to beta-CGRP. However, the pharmacological actions of CGRP in the gastrointestinal system are complex, comprising both excitatory and inhibitory actions on gastrointestinal motility, which is explained by the multiple sources of CGRP in the gut. While CGRP released by EPANs (mostly alpha-CGRP) inhibits gastrointestinal motility under some pathological conditions, CGRP from IPANs (beta-CGRP) plays a physiological role in initiating peristaltic (propulsive) motility and secretion in the intestine, leading to diarrhea, which in experimental animals can be stopped by CGRP antagonists [17]. Taken together, there is substantial evidence from studies in laboratory animals that CGRP can stimulate propulsive motor activity and secretion; however, species differences between laboratory animals and humans are always possible, and the number of experimental studies in isolated preparations of the human intestine is limited (see reference [18] for review). The development of diarrhea after CGRP infusion in human volunteers [7], the appearance of constipation as the main adverse event of treatment with CGRP antagonists [8,18], or the development of diarrhea as a symptom of patients with neuroendocrine tumors expressing CGRP [19] support a role of CGRP in promoting intestinal motility in humans [18]. Moreover, our previous finding that beta-CGRP is specifically increased in COVID-19 patients suffering from diarrhea [10] conceptually concurs with the current results showing a decrease in beta-CGRP in patients developing constipation on mAb treatment and indicates a role of this isoform in the physiology of intestinal motility. The beta-CGRP isoform plays a key protective role in the homeostasis of the alimentary tract, as shown by a number of experimental data and by its early decrease in patients with inflammatory bowel disease [11,18,20,21]. Chronic blockade of gut beta-CGRP by mABs could theoretically contribute to modifying the physiological homeostasis of the intestinal mucosa in patients with inflammatory bowel diseases and, considering that there is a comorbidity between migraines and IBD [22], the status of IBD in patients with mAbs should be closely monitored.

### 4.2. Limitations

Our study has some limitations. First, the low proportion of patients developing constipation on mAbs makes it difficult to draw definite conclusions. In our defense, the observed rate of constipation with mAbs in our series is the average of those reported in the literature [8,18], and we had to collect a high number of migraine patients, before and after mAbs, to analyze this point. Even though further studies including a higher number of subjects are necessary to confirm these results, our data are a preliminary proof of concept on the pathophysiology of constipation on mAbs and fit well with the proposed action of beta-CGRP in the gut. The same is true for gender; the very low proportion of men makes it difficult to extrapolate our results to this sex. Second, though there were no significant differences in the rates and profiles of comorbidities and consumed treatments between the patients exhibiting constipation and those who did not, an influence of these variables on CGRP levels is still possible. For instance, even though all our blood samples were obtained without having taken symptomatic medication the previous day, we cannot rule out analgesic overuse as a potential confounding factor. Finally, we cannot rule out the role of other molecules in the pathophysiology of constipation seen with mAbs. Amylin could be one candidate, as constipation occurs more frequently with erenumab, which has an affinity for amylin receptors, than with mAbs antagonizing circulating CGRP [8,18], but results from experimental models [23] or in clinical trials with amylin agonists—with no evidence of diarrhea as an adverse event [24,25]—are controversial regarding its action on intestinal motility.

## 5. Conclusions

This study explores the role of the two isoforms of CGRP in the pathophysiology of emergent constipation with mAbs and provides evidence supporting that antagonism on the alpha-CGRP isoform mainly explains their efficacy on migraine prevention but not in the development of constipation. The selective reduction in beta-CGRP circulating levels three months after treatment with mAbs lends weight to a role for beta-CGRP blockade in the development of constipation and is a further example of the role of this neuropeptide in the physiology of the gastrointestinal system. These results should be confirmed in future studies including a higher number of patients but are an example of the complex, bidirectional molecular mechanisms underlying the pathophysiology of a variety of diseases involving the gut–brain axis.

## Figures and Tables

**Table 1:** Basal characteristics of our migraine patients (n = 133).

Mean age, range	47.7 ± 10.7, 19–69 years
Women (%)	128 (96.2%)
Diagnosis	
Chronic migraine	111 (84.1%)
High-frequency episodic migraine	22 (15.9%)
History of aura	42 (31.6%)
Analgesic overuse	114 (83.5%)
Main comorbidities	
Anxiety/depression	70 (52.6%)
Obesity	25 (18.9%)
Fibromyalgia	23 (17.3%)
Arterial hypertension	23 (17.3%)
Preventive treatments	
Oral preventatives	101 (75.9%)
OnabotulinumtoxinA	47 (35.3%)
Efficacy measures at month 3	
Reduction in monthly headache days	−11.64 (*p* < 0.0001)
Reduction in monthly migraine days	−10.33 (*p* < 0.0001)
Monthly headache response (<50%) rate	81 (60.9%)
Monthly headache response (<50%) rate	87 (65.4%)
Constipation at month 3	12 (9.0%)

**Table 2:** Comparison between patients with versus without emergent constipation. Bold indicates significant values.

Variable	Constipation	No Constipation	*p*
Mean age ± SD	51.25 ± 11.33	47.29 ± 10.56	0.330
Women (%)	11/12 (91.7%)	117/121 (96.7%)	0.382
Chronic migraine	11/12 (91.7%)	100/121 (82.6%)	0.422
HFEM	1/12 (8.3%)	21/121 (17.93%)	0.422
History of aura	3/12 (25%)	39/121 (32.2%)	0.607
Analgesic overuse	11/12 (91.7%)	103/121 (85.1%)	0.537
Anxiety/depression	10/12 (83.3%)	60/121 (49.6%)	**0.026**
Obesity	2/12 (16.7%)	23/121 (19%)	0.833
Fibromyalgia	2/12 (16.7%)	21/121 (17.3%)	0.952
Arterial hypertension	1/12 (8.3%)	2/121 (18.2%)	0.390
Oral preventives	10/12 (83.3%)	91/121 (75.2%)	0.530
OnabotulinumtoxinA	7/12 (58.3%)	40/121 (33.1%)	0.081
Response (headache days)	5/12 (41.7%)	76/121 (62.8%)	0.152
Response (migraine days)	5/12 (41.7%)	82/121 (67.8%)	0.070
PGIC scale score	4.25 ± 1.77	2.67 ± 1.54	**0.002**

## Data Availability

Data are contained within the article.

## Abbreviations

ELISA	Enzyme-linked immunosorbent assay
CGRP	Calcitonin gene-related peptide
CM	Chronic migraine
HFEM	High-frequency episodic migraine
IQR	Interquartile range
mAb	CGRP monoclonal antibodies
PGIC	Patient Global Impression of Change
SD	Standard deviation
